# A Randomized Trial on the Benefits of Refitting Symptomatic Contact Lens Wearers with Daily Disposable Lenses

**DOI:** 10.3390/jcm14186575

**Published:** 2025-09-18

**Authors:** José Miguel Sánchez-Ruiz, A. Eusebio López-Hernández, Youssef Abidi, Johnny Di Pierdomenico, Diego García-Ayuso

**Affiliations:** 1 Grupo de Investigación Optometría Clínica y Experimental, Facultad de Óptica y Optometría, Universidad de Murcia, 30100 Murcia, Spain; jmsanchez@um.es (J.M.S.-R.); antonioeusebio.lopezh@um.es (A.E.L.-H.); youssef.abidi@um.es (Y.A.); 2 Grupo de Investigación Oftalmología Experimental, Departamento de Oftalmología, Optometría, Otorrinolaringología y Anatomía Patológica, Facultad de Medicina, Instituto Murciano de Investigación Biosanitaria Pascual Parrilla-IMIB, Universidad de Murcia, 30120 Murcia, Spain

**Keywords:** contact lens discomfort, daily disposable lenses, ocular surface, refitting, contact lens wearers

## Abstract

**Background/Objective**: Contact lens discomfort (CLD) is a prevalent issue affecting up to 50% of wearers and contributes to discontinuation in approximately 30% of cases. This study aimed to evaluate the impact of refitting symptomatic monthly replacement contact lens (CL) wearers with a new daily, disposable contact lens (Kalifilcon A DDCL) and to compare its effectiveness against a placebo CL. **Methods**: Seventy-nine symptomatic wearers (CLDEQ-8 ≥ 12; mean age 27.07 ± 8.38 years; 77% female) were recruited and randomly assigned to wear either Kalifilcon A DDCLs or placebo CLs. Participants were assessed at baseline and after one month of CL wear. Subjective measures included the CLDEQ-8 and the NEI VFQ-25 questionnaire. Objective assessments included tear film stability and ocular surface health. Statistical analysis was performed using paired *t*-tests and repeated-measures ANOVA. Bonferroni correction was applied for multiple comparisons, and results were reported with effect sizes (Cohen’s d) and 95% confidence intervals. Statistical significance was set at *p* < 0.05. **Results**: Kalifilcon A DDCL wearers showed a significant reduction in CLDEQ-8 scores (18.5 ± 4.6 to 10.8 ± 8.5; *p* < 0.005; Cramer’s V: 0.594; 95% CI: 13.35–15.34), with only 33% remaining symptomatic. Vision-related quality of life improved (75.83 ± 8.0 to 83.5 ± 8.6; *p* < 0.005; Cramer’s V: 0.977; 95% CI: 78.54–80.81), especially in ocular pain (*p* < 0.001; Cramer’s V: 0.755; 95% CI: 54.73–58.88), role difficulties (*p* < 0.001; Cramer’s V: 0.812; 95% CI: 35.12–42.77), and color vision (*p* < 0.05; Cramer’s V: 0.575; 95% CI: 93.07–95.63). Compared to the placebo CL, Kalifilcon A DDCLs led to greater improvements in comfort (*p* < 0.0001; Cramer’s V: 0.329; 95% CI: 7.22–7.74), visual acuity (*p* < 0.0001; Cramer’s V: 0.267; 95% CI: 7.38–7.85), and satisfaction (*p* = 0.005; Cramer’s V: 0.208; 95% CI: 7.70–8.18). Tear film stability also improved significantly (*p* < 0.05), with no changes observed in the placebo group. **Conclusions**: Refitting symptomatic CL wearers with Kalifilcon A DDCLs significantly improves comfort, reduces CLD symptoms, and enhances vision-related quality of life. These results support its use as a beneficial alternative to monthly CLs in symptomatic individuals.

## 1. Introduction

Contact lenses (CLs) offer a convenient and attractive alternative to traditional eyeglasses for the optical correction of refractive error, providing a heightened level of satisfaction and enhancing the overall quality of life [1,2]. Despite the progress made in CL comfort and safety, CL discomfort (CLD) is estimated to affect up to 50% of CL wearers [3]. Moreover, up to 30% of CL wearers permanently discontinue CL wear because of CLD [3,4]. CLD is a complex issue [5] that has been defined by the Tear Film and Ocular Surface (TFOS) International Workshop as “a condition characterized by episodic or persistent adverse ocular sensations related to CL wear, either with or without visual disturbance, resulting from reduced compatibility between the CLs and the ocular environment, which can lead to decreased wearing time and discontinuation of CL wear” [6]. It is important to identify symptomatic CL wearers and implement strategies to prevent CL dropout, which has the potential to negatively affect their quality of life [1,2,7].

CLD is typically diagnosed based on the patient’s reported symptoms rather than through observation of physical signs. Therefore, questionnaires that assess the parameters of discomfort specific to this condition are useful tools in the diagnostic process. Of the available questionnaires, the Contact Lens Dry Eye Questionnaire-8 (CLDEQ-8) is traditionally used as the questionnaire of choice to identify symptomatic CL users [5,8]. One of the most common approaches to prevent or at least reduce CLD is to increase the frequency of CL replacements [9,10,11]. The reduced presence of deposits on the CL surface and improved wettability, coupled with the lack of the need to use care systems (which can also contribute to CLD), make CL replacement with a daily disposable CL (DDCL) one of the most effective solutions for reducing CLD symptoms or complications [4,9,11]. Nevertheless, it is essential to consider the possibility of a placebo effect when conducting such studies [10]. Therefore, it is crucial to demonstrate the efficacy of the treatment, considering the potential influence of the placebo effect.

Quality of life is an essential aspect of refractive care. A significant objective of CL fitting is to attain optimal vision and comfort, which ultimately results in patient satisfaction and an improved quality of life [7]. The World Health Organization defines quality of life as “an individual’s perception of his or her position in life in the context of the culture and value systems in which he or she lives and in relation to his or her goals, expectations, standards and concerns” [12]. The 25-item version of the National Eye Institute Visual Function Questionnaire (NEI VFQ-25) is a validated questionnaire [13] that has been used to assess the quality of life in several eye conditions [14,15,16,17].

This study aimed to evaluate the efficacy of refitting symptomatic CL wearers with a new DDCL (Kalifilcon A) in improving comfort and enhancing vision-related quality of life.

## 2. Materials and Methods

This was a single-center, single-blinded, prospective, randomized crossover study, allowing each subject to serve as their own control. This study was approved by the Clinical Research Ethics Committee (CEIC) of the University of Murcia and complied with the Tenets of the Declaration of Helsinki.

### 2.1. Participants

The Spanish version of the CLDEQ-8 questionnaire [18] was distributed to monthly CL wearers who had a minimum of six months of CL wear experience, and those who were symptomatic (CLDEQ-8 ≥ 12) were invited to participate in this study. The inclusion criteria were age between 18 and 40 years, spherical equivalent refraction between −1.00 and −6.00 D, astigmatism ≤ −0.75 D, and best-corrected visual acuity of 20/20 or better. The exclusion criteria were CL wear other than monthly replacement (including overnight use), dry eye disease according to the TFOS DEWS II diagnostic criteria [19], and active ocular allergies, ocular diseases, history of previous eye surgery, topical medication, or systemic diseases contraindicating CL wear. Additionally, asymptomatic CL wearers were excluded according to the CLDEQ-8 (score < 12 points) questionnaire.

**Figure 1:**
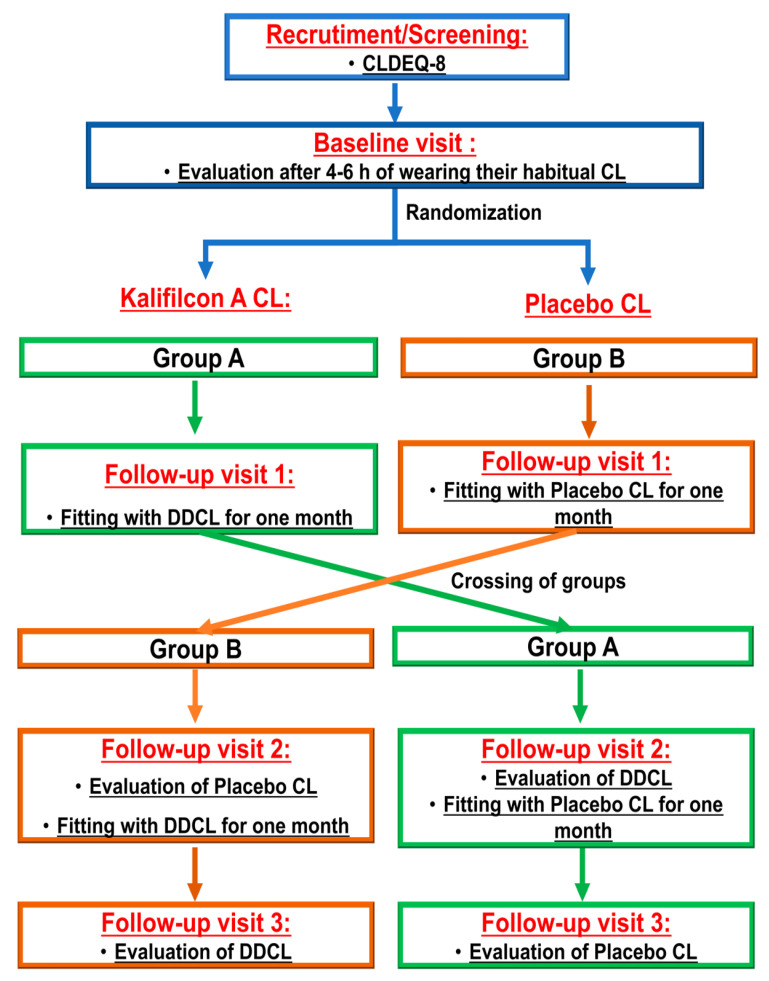
Study design layout. The blue boxes represent the entire group, while the green and brown colors represent the two groups into which the subjects are divided. The division into these two groups is randomized in order to ensure an equitable randomization of CL wear.

Participants were randomized into two groups (two sequences). One group was refitted with Kalifilcon A CLs (ULTRA^®^ One Day, Bausch & Lomb Inc., Rochester, NY, USA), while the other was refitted with a masked pair of their habitual CLs (Figure 1) to balance potential period effects.

### 2.2. Procedure

All optometric measurements were conducted by the same optometrist in the same order to minimize potential variability resulting from examiner subjectivity.

Following recruitment, all the selected participants attended four visits.

Baseline visit: Participants were asked to attend a visit 4–6 h after wearing their habitual CLs. At this visit, their habitual CLs were assessed, and participants were provided with information about their experience of use. They completed the CLDEQ-8 [5,8,18] and the NEI VFQ-25 [13] questionnaires to assess symptomatology and vision-related quality of life during CL wear, and a series of questions regarding their subjective satisfaction with the CLs used [1]. Visual acuity (VA) and auto-tear break-up time were evaluated using the Medmont E300, version 6.1 (Medmont, medmont.com.au). This device provides three values for each measurement: tear film surface quality (TFSQ), TFSQ area (A-TFSQ), and auto-tear break-up time (TBUT). TFSQ is a previously validated algorithm [20]. Finally, participants removed their CLs to perform the necessary tests for the fitting study. These tests included corneal topography using the Medmont E300 and eye health evaluation using the Efron grading scale. At the end of this visit, CLs were ordered from the manufacturers.

Visit 1: Half of the participants were randomly selected and administered DDCLs for one month (Kalifilcon A, ULTRA One Day, Bausch and Lomb). The other half of the participants were provided with a new pair of their usual monthly CLs masked in a CL case and were informed that they were from a new manufacturer. CL fitting, TFSQ, A-TFSQ, TBUT, and VA were assessed after 25 min of wear, and the participants were instructed to wear the new pair of CLs according to their usual routine and were provided with standardized guidelines for proper handling and cleaning, to be followed until their next visit. To avoid introducing an additional variable into this study, they were asked to continue using their habitual multipurpose solution for cleaning and storage.

Visit 2: Participants were cited one month after visit 1 to assess CL fitting. During this visit, participants answered the CLDEQ-8, the NEI VFQ-25, and their subjective satisfaction with the CL questionnaires. For clinical evaluation, the same protocol as that described in the first visit was applied.

During this visit, the groups were crossed, with participants who had used DDCLs given their usual monthly CLs masked, and vice versa. Once more, CL fitting and all clinical evaluation tests were assessed after 25 min of wear, and participants were requested to continue using the CLs provided to them.

Visit 3: One month following the second visit, participants were cited to assess CL fitting. The same protocol as that described for the first visit was used. The participants completed this study upon finishing the evaluation of this visit. By the study’s conclusion, all participants had worn two different CLs for one month each. Finally, the participants were requested to indicate which of the two CLs they preferred in terms of comfort and vision.

### 2.3. Questionnaires

The NEI-VQF-25 is a validated and comprehensive questionnaire designed to assess the impact of vision impairment on an individual’s quality of life [13]. It includes 25 items divided into several subscales, each targeting different aspects of vision-related quality of life: general health, general vision, ocular pain, near activities, distance activities, social functioning, mental health, role difficulties, dependency, driving, color vision, and peripheral vision. Each item on the VQF-25 is rated on a scale, with higher scores indicating greater difficulties and a more significant impact on quality of life.

The CLDEQ-8 is a concise questionnaire that has been validated to measure the frequency and severity of dry eye symptoms in CLs wearers [8]. This questionnaire, consisting of eight items, focuses on common symptoms, such as dryness, discomfort, and visual disturbances, which are often intensified by the use of CLs. Each item on the CLDEQ-8 was rated based on frequency and severity, with higher scores indicating more significant symptoms and a greater impact on the individual’s comfort and satisfaction with CL wear.

The questionnaire regarding their subjective satisfaction with the CLs consisted of seven questions rated on a 0–10 scale (higher scores indicating greater satisfaction with the CLs) related to handing for application, handling for removal, comfort, vision clarity, overall satisfaction with comfort, overall satisfaction with vision clarity, and overall satisfaction with these CLs to evaluate the participants’ subjective satisfaction with the CLs [1].

### 2.4. Statistical Analysis

Data were analyzed using the Statistical Package for Social Sciences software version 28 (SPSS, International Business Machine Corp. IBM, Chicago, IL, USA). Results are presented as mean ± standard deviation (SD). Paired t-tests were applied to assess within-subject differences between CL types. A Bonferroni correction was applied. Given the crossover design, in addition to paired comparisons, we performed repeated-measures ANOVA to account for within-subject correlation and to explore possible period and sequence effects. For each outcome, the CL was entered as a within-subject factor. Where homogeneity of variances was violated, robust tests (Welch) were applied. Post hoc comparisons were corrected using Tukey’s procedure. For all key comparisons, effect sizes were calculated to provide an estimate of the magnitude of differences. For continuous outcomes (tear film parameters), Cohen’s d was used. For categorical outcomes (CLDEQ-8, experience of use, and NEI VFQ-25 subscales), Cramer’s V was calculated. Effect sizes were interpreted as small (0.1–0.3), moderate (0.3–0.5), or large (>0.5), and results are reported together with 95% confidence intervals (CI). Univariate analyses (chi-squared test) were performed to describe the relationship between the CLDEQ-8 test and CL type. Although no a priori sample size calculation was performed, a post hoc analysis indicated that this study (*n* = 79) had >80% power to detect the observed clinically significant difference in CLDEQ-8 scores between Kalifilcon A and placebo CL at α = 0.05. The significance level was set at *p* < 0.05.

## 3. Results

A total of 79 symptomatic monthly replacement CL wearers (CLDEQ-8 ≥ 12) were recruited for this study. The mean age ± SD of the participants was 27.07 ± 8.38 (range 19–40). Of the 79 participants, 77% were female and 23% were male. The demographic and baseline information are summarized in Table 1.

### 3.1. Kalifilcon A CL Refitting

**Figure 2:**
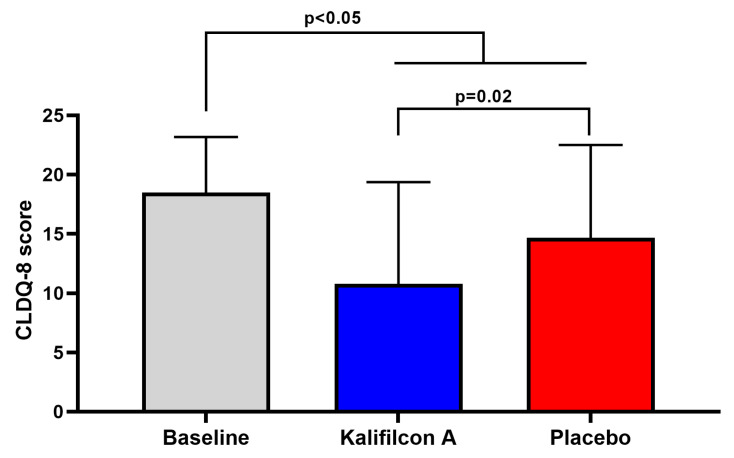
Mean values of the scores obtained in the CLDEQ-8 test. We found a decrease in the total score both when participants were readapted with the placebo CL (red) and with the Kalifilcon A CL (blue). However, the decrease in the score is significantly less when participants are readapted with the Kalifilcon A CL compared to the placebo CL. Student’s *t*-test.

**Figure 3:**
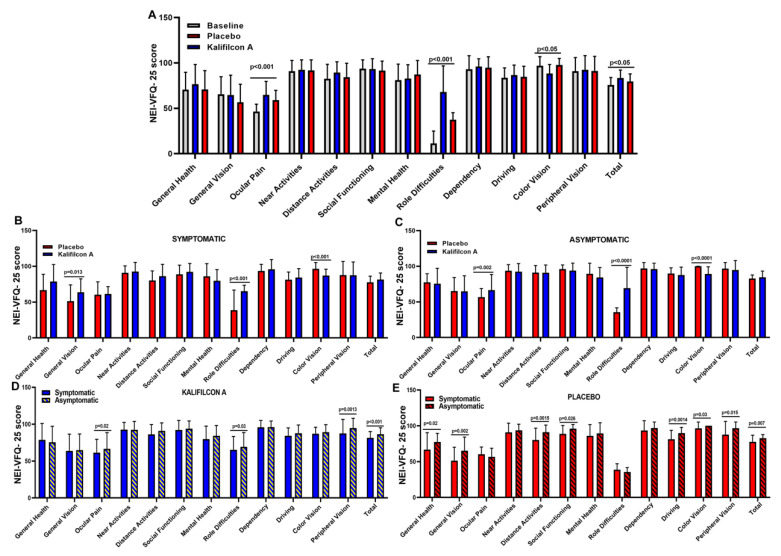
Mean values of the score obtained in the NEI-VFQ-25 test, dividing the participants according to the CL used (**A**), according to result obtained in the CLDEQ-8 test in symptomatic (**B**), or non-symptomatic (**C**), and according to the results obtained in the CLDEQ-8 and with which CL they have been refitted, Kalifilcon A DDCLs (**D**) or Placebo CLs (**E**). Student’s *t*-test.

After one month of wearing Kalifilcon A DDCL, the mean CLDEQ-8 score showed a statistically and clinically significant reduction compared to baseline (10.8 ± 8.5 and 18.5 ± 4.6; *p* < 0.005; Cramer’s V: 0.594; 95% CI: 13.35–15.34; Figure 2). This reduction in symptomatology was also reflected in a statistically significant improvement in vision-related quality of life as measured using the NEI VFQ-25 questionnaire. The overall score was 75.83 ± 8.0 at baseline and 83.5 ± 8.6 with the Kalifilcon A DDCLs (*p* < 0.005; Cramer’s V: 0.977; 95% CI: 78.54–80.81; Figure 3). More specifically, participants refitted with Kalifilcon A DDCLs indicated better feedback on ocular pain (*p* < 0.001; Cramer’s V: 0.755; 95% CI: 54.73–58.88), role difficulties (*p* < 0.001; Cramer’s V: 0.812; 95% CI: 35.15–42.77), and color vision (*p* < 0.05; Cramer’s V: 0.575; 95% CI: 93.07–95.63).

A comparison of CLDEQ-8 results at baseline and one month following Kalifilcon A CL wear revealed that while 100% were symptomatic at the beginning of this study, only 33% remained symptomatic after one month, while 67% were classified as asymptomatic.

To assess whether there is a direct relationship between vision-related quality of life and symptoms during CL wear, NEI VFQ-25 scores were compared between symptomatic and asymptomatic participants after refitting with Kalifilcon A DDCL. The overall score showed a significantly higher vision-related quality of life (*p* < 0.01; Cramer’s V: 977; 95% CI: 78.54–80.81) in the asymptomatic (86.5 ± 8.6) compared to symptomatic (81.5 ± 8.6) participants (Figure 3D,E). More specifically, asymptomatic participants showed a significant improvement when refitted with Kalifilcon A DDCLs in the ocular pain (*p* = 0.02; Cramer’s V: 0.755; 95% CI: 54.73–58.88)), role difficulties (*p* = 0.03; Cramer’s V: 0.812; 95% CI: 35.12–42.77), and peripheral vision (*p* = 0.0013; Cramer’s V: 0.140; 95% CI: 89.47–93.44) of the NEI VFQ-25 subscales.

**Figure 4:**
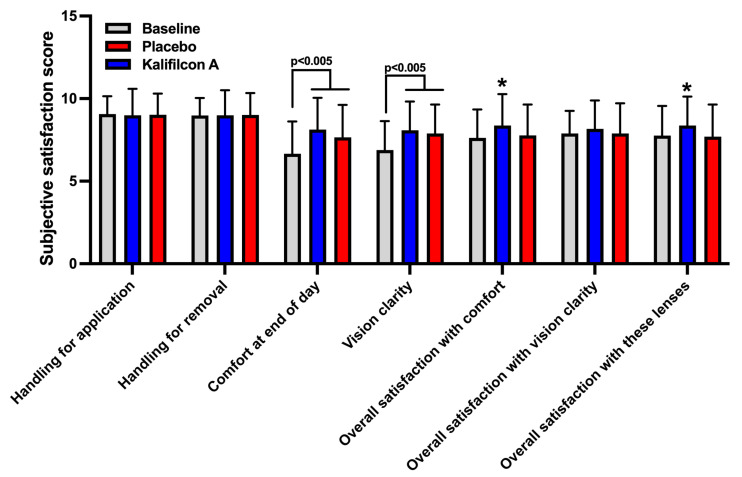
Mean values of the score obtained in the Subjective Satisfaction with the CLs test. Participants found a significant improvement in the readaptation of both CLs in overall comfort during CL wear and sharpness of vision with the CLs. However, they only found a significant improvement in the evaluation of overall satisfaction with comfort and overall satisfaction with the CLs when they were readapted with the Kalifilcon A DDCL. * = statistical difference with baseline and placebo CL *p* < 0.001. Student’s *t*-test.

Participants rated their experience with Kalifilcon A DDCLs using a 10-item standardized questionnaire [1]. The results demonstrated a clear and significant improvement in overall comfort assessment (*p* < 0.0001; Cramer’s V: 0.329; 95% CI: 7.22–7.74), visual acuity (*p* < 0.0001; Cramer’s V: 0.267; 95% CI: 7.38–7.85), overall satisfaction with the CLs (*p* = 0.005; Cramer’s V: 0.305; 95% CI: 7.68–8.16), and general satisfaction with the CLs (*p* = 0.0004; Cramer’s V: 0.208; 95% CI: 7.70–8.18) among patients refitted with Kalifilcon A DDCLs compared to the previous/baseline results (Figure 4).

**Figure 5:**
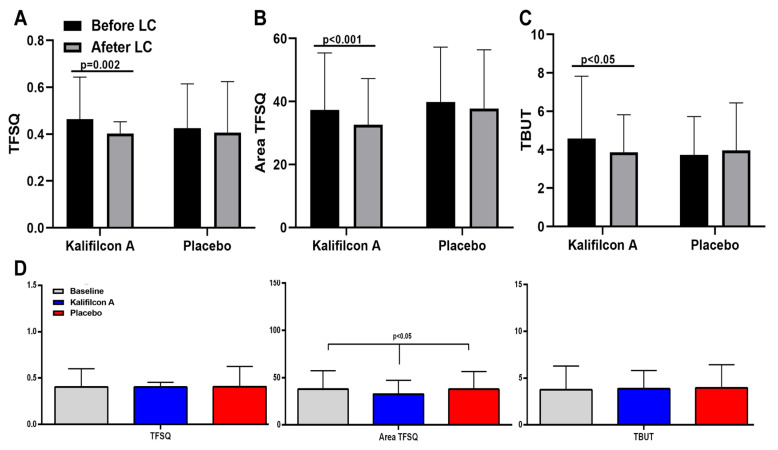
Mean values of tear stability. An improvement is observed in the values of (**A**) automated tear film surface quality break-up time (TFSQ); (**B**) area of the TFSQ; and (**C**) tear break-up time (TBUT) between the first day and the last day after readaptation with the Kalifilcon A DDCL. (**D**) Comparing the baseline values with those obtained after one month of readaptation, we found a statistically significant improvement in the area of the TFSQ in patients readapted with the Kalifilcon A DDCL. Student’s *t*-test.

The clinical evaluation of the auto-tear break-up time showed a significant reduction in TFSQ (*p* = 0.002; Cohen’s d: 0.184; 95% CI: 0.34–0.58), A-TFSQ (*p* < 0.001; Cohen’s d: −0.055; 95% CI: 33.06–41.56), and TBUT (*p* < 0.05; Cohen’s d: 0.216; 95% CI: 3.81–4.75) after one month refitted with Kalifilcon A DDCLs (Figure 5).

Furthermore, no discernible alterations in visual acuity or ocular health (Efron grading scale) were observed following either CL wear compared to those observed at the baseline visit.

### 3.2. Placebo CL Refitting

After one month of wearing the Placebo CLs, the mean total score on the CLDEQ-8 questionnaire showed a statistically significant reduction from the baseline (15.0 ± 7.5 compared to 18.5 ± 4.6; *p* < 0.05; Cramer’s V: 0.594; 95% CI: 13.35–15.34; Figure 2). Considering that 100% of the participants were symptomatic at the beginning of this study, after one month of using the placebo CLs, 40% became non-symptomatic, indicating a placebo effect.

The placebo effect on symptomatology was also reflected in a significant improvement in vision-related quality of life as measured by the NEI VFQ-25 questionnaire. The overall score was 75.83 ± 8.0 at baseline and 79.6 ± 8.1 with the Placebo CLs (*p* = 0.003; Cramer’s V: 0.977; 95% CI: 78.54–80.81; Figure 3). Similar to the results obtained with the Kalifilcon A DDCL, when the participants were refitted with Placebo CLs, they provided better feedback in ocular pain (*p* < 0.001; Cramer’s V: 0.755; 95% CI: 54.73–58.88), role difficulties (*p* < 0.001; Cramer’s V: 0.812; 95% CI: 35.12–42.77), and color vision (*p* < 0.05; Cramer’s V: 0.575; 95% CI: 93.07–95.63).

Moreover, in the Placebo CLs, there is a direct relationship between vision-related quality of life and symptoms during CL wear. The overall score of the NEI VFQ-25 scores was compared between symptomatic and asymptomatic participants after refitting with Placebo CLs, showing a significantly higher vision-related quality of life (*p* = 0.007; Cramer’s V: 0.977; 95% CI: 78.54–80.81) in the asymptomatic (82.7 ± 4.9) compared to symptomatic (77.8 ±9.1) participants (Figure 3). More specifically, asymptomatic participants observed a significant improvement when refitted with Placebo CLs in general health (*p* = 0.02; Cramer’s V: 0.198; 95% CI: 70.05–75.31), general vision (*p* = 0.002; Cramer’s V: 0.359; 95% CI: 59.63–64.92), distance activities (*p* = 0.0015; Cramer’s V: 0.243; 95% CI: 83.58–87.32), social functioning (*p* = 0.026; Cramer’s V: 0.432; 95% CI: 91.54–94.21), driving (*p* = 0.0014; Cramer’s V: 0.164; 95% CI: 83.54–86.57), color vision (*p* = 0.03; Cramer’s V: 0.575; 95% CI: 93.07–95.63), and peripheral vision (*p* = 0.015; Cramer’s V: 0.140; 95% CI: 89.47–93.44) of the NEI VFQ-25 subscales.

In the subjective rate of their experience with the Placebo CLs, the participants indicated only a significant improvement in the overall comfort assessment (*p* < 0.005; Cramer’s V: 0.329; 95% CI: 7.22–7.74) and visual acuity (*p* < 0.005; Cramer’s V: 0.267; 95% CI: 7.38–7.85), compared to the previous/baseline results (Figure 4).

In contrast with the results obtained with Kalifilcon A DDCL, the clinical evaluation of the auto-tear break-up time after one month refitted with Placebo CLs showed no differences in TFSQ (*p* = 0.56; Cohen’s d: −0.078; 95% CI: 0.37–0.41), A-TFSQ (*p* = 0,38; Cohen’s d: −0.309; 95% CI: 32.55–37.99), and TBUT (*p* < 0.55; Cohen’s d: 0.047; 95% CI: 3.47–4.18) compared to baseline values (Figure 5).

**Figure 6:**
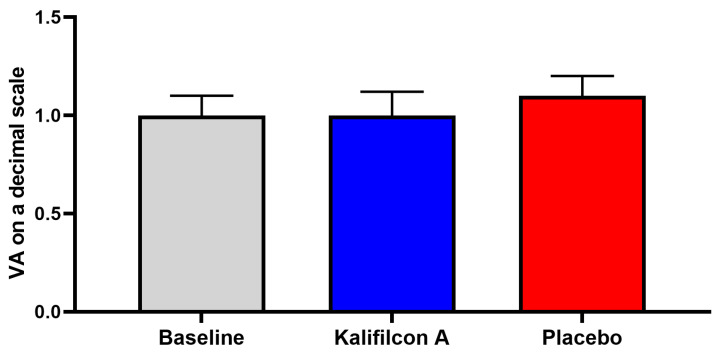
Mean values of visual acuity on a decimal scale. VA was measured with their CL (baseline) and after the readaptation of each CL and no statistical differences were found. Student’s *t*-test *p* > 0.05.

Furthermore, no discernible alterations in visual acuity (Figure 6) or ocular health (Efron grading scale) were observed following either CL wear compared to those observed at the baseline visit.

### 3.3. Kalifilcon A Versus Placebo CL Refitting

The mean total score obtained with the CLDEQ-8 questionnaire demonstrated a statistically and clinically significant reduction when the participants were refitted with Kalifilcon A DDCLs in comparison to Placebo CLs (10.8 ± 8.5 and 15.0 ± 7.5; *p* = 0.02; Cramer’s V: 0.594; 95% CI: 13.35–15.34; Figure 2). This reduction in symptomatology was also reflected in a statistically significant improvement in vision-related quality of life as measured using the NEI VFQ-25 questionnaire. The overall score was 79.67 ± 8.1 (95% CI: 78.57–80.81) with Placebo CLs and 83.5 ± 8.6 with the Kalifilcon A DDCLs (*p* = 0.004; Cramer’s V:0.977; 95% CI: 81.46–85.45; Figure 3). More specifically, participants refitted with Kalifilcon A DDCLs indicated better feedback on ocular pain (*p* < 0.001; Cramer’s V: 0.755; 95% CI: 54.73–58.88), role difficulties (*p* < 0.001; Cramer’s V: 0.812; 95% CI: 35.12–42.77), and color vision (*p* < 0.05; Cramer’s V: 0.575; 95% CI: 93.07–95.63).

To assess whether there was a direct relationship between vision-related quality of life and symptoms depending on what CLs the participant wear, symptomatic and asymptomatic participants’ NEI VFQ-25 scores were compared after refitting with Kalifilcon A DDCLs or Placebo CLs. The overall scores showed no statistical difference in vision-related quality of life between symptomatic (*p* = 0.32) and asymptomatic (*p* = 0.09) participants (Figure 3). However, in the NEI VFQ-25 subscales, symptomatic participants indicated a significant improvement in general vision (*p* = 0.013) and role difficulties (*p* < 0.001) when they were refitted with Kalifilcon A DDCL. Furthermore, the asymptomatic participants indicated a significant improvement in ocular pain (*p* = 0.002; Cramer’s V; 0.755; 95% CI: 54.73–58.88) and role difficulties (*p* < 0.0001; Cramer’s V: 0.812; 95% CI: 35.12–42.77) when they were refitted with Kalifilcon A DDCL. However, both symptomatic (*p* < 0.001; Cramer’s V: 0.575; 95% CI: 93.07–95.63) and asymptomatic (*p* < 0.0001; Cramer’s V: 0.575; 95% CI: 93.07–95.63) participants indicated a significant improvement in color vision subscales when they were refitted with Placebo CLs.

In the results of the evaluation of the subjectively rated experience of wear, the CLs demonstrated a clear and significant improvement in general satisfaction with comfort (*p* = 0.003; Cramer’s V: 0.329; 95% CI: 7.22–7.74) and overall satisfaction with the CL (*p* = 0.0002; Cramer’s V: 0.208; 95% CI: 7.70–8.18) among patients refitted with Kalifilcon A DDCLs compared to Placebo CLs (Figure 4).

The clinical evaluation of the auto-tear break-up time showed only a significant reduction in A-TFSQ (*p* < 0.05; Cohen’s d: −0.309; 95% CI: 32.55–37.99) after one month refitted with Kalifilcon A DDCLs in comparison with Placebo CLs (Figure 5).

Furthermore, no discernible alterations in visual acuity (Figure 6) or ocular health (Efron grading scale) were observed following either CL wear.

The ANOVA revealed a significant main effect of CL type on overall discomfort, dryness, blurry vision, and related items (all *p* < 0.001). The results indicated that Kalifilcon A DDCLs consistently yielded lower symptom scores in comparison to placebo and baseline CLs. Subsequent post hoc tests confirmed significant pairwise differences (Tukey-adjusted, *p* < 0.05).

ANOVA analyses demonstrated significant enhancements in several NEI VFQ-25 subscales when wearing Kalifilcon A DDCLs in comparison to baseline, particularly in general vision, ocular pain, role difficulties, mental health, and color vision (all *p* < 0.05). No significant differences were identified for driving, near activities, or dependency subscales (*p* > 0.5). The observed pattern of results suggests that Kalifilcon A DDCLs provide a measurable improvement in quality-of-life domains related to comfort and visual function.

It was observed that there were no significant differences between CL types regarding ease of handling and insertion/removal (*p* > 0.5). However, Kalifilcon A DDCLs were found to demonstrate significantly higher comfort, sharpness, and satisfaction scores in comparison to baseline (*p* < 0.01). Placebo CLs exhibited intermediate results. The differences in satisfaction with sharpness were found to be statistically significant only between Kalifilcon A DDCLs and baseline (*p* = 0.029).

## 4. Discussion

The aim of this study was to fit symptomatic CL wearers, who are at risk of CL dropout, with a modern DDCL to evaluate whether this intervention could lessen the symptoms they experience, enhance their overall CL experience, and ultimately reduce the likelihood of future dropout. The findings of this study demonstrate that following the refitting of symptomatic wearers with Kalifilcon A DDCL, 67% become asymptomatic. This has the potential to directly impact clinical practice by reducing CL dropout rates.

The prescription of DDCLs is a common practice among practitioners with the aim of improving the comfort of CL wearers [9,21] and decreasing the dropout rates of CL wearers. This is in agreement with the results of this study showing a significant improvement in CLDEQ-8 scores after fitting with Kalifilcon A DDCL. Specifically, the mean difference obtained between the baseline and the CLs under study was 7.7, which is above the threshold for clinical significance (≥3; [22]) and above the differences observed in similar studies [9,23]. The observed differences could be explained by the type of CLs used, with Toric CLs being used in one of these studies, or by the size of the sample analyzed, which was much larger in this study. Regarding the placebo effect, the mean difference obtained was 3.5, a value that is marginally above the threshold for a clinically significant difference, although lower than that found when refitting with Kalifilcon A DDCL. A statistically significant difference was observed between the results obtained with Kalifilcon A DDCLs and Placebo CLs. This finding is in contrast with a previous study in which, although the experimental group demonstrated superior results in comparison to the placebo, these differences were not statistically significant [10]. It is noteworthy that these discrepancies may be attributable to the considerably larger sample size employed in this study.

The impact of CL wear on vision-related quality of life has been previously examined using the NEI VFQ-25 in myopic patients, with a view to comparing the performance of monofocal versus multifocal CLs [24], and following CL fitting for the management of irregular cornea [25]. However, to the best of our knowledge, this study is the first to analyze vision-related quality of life after CL refitting using the NEI VFQ-25. The findings of this study demonstrate the impact of refitting on vision-related quality of life, which exhibited a significant enhancement. Notably, substantial improvements were observed in the subscales of ocular pain, role difficulties and color vision. While the Placebo CLs also led to an improvement in vision-related quality of life, this was more pronounced with Kalifilcon A DDCL. Finally, vision-related quality of life was consistently superior in non-symptomatic subjects who received Kalifilcon A DDCL, as compared to those who received Placebo CLs.

The Kalifilcon A DDCLs performed better in terms of subjective ratings for comfort and overall satisfaction compared to placebo, whereas handling when putting in and for removal were similar for both.

This study demonstrated an enhancement in tear film stability subsequent to the refitting with Kalifilcon A DDCLs. Specifically, the clinical evaluation of auto-tear break-up time exhibited enhancement following refitting with Kalifilcon A DDCLs in comparison to the habitual CLs, a phenomenon that was not observed in the placebo group. Furthermore, a substantial reduction was observed in both the TFSQ and A-TFSQ. CL wear can adversely affect the tear film and anterior ocular surface, leading to reduced wearing times and, eventually, to CL discontinuation [26]. Indeed, it is a recognized fact that individuals who wear CLs are more prone to experiencing symptoms of dry eye [27], a factor that can directly impact the symptomatology during CL wear. The association between CL discomfort and precorneal tear film stability has been previously demonstrated [28]. Consequently, the observed enhancement in tear film stability can be considered a potential underlying factor contributing to the mitigation of symptomatology and the enhancement of vision-related quality of life post-refitting with Kalifilcon A DDCL. The large effect sizes observed for the CLDEQ-8 total score, ocular pain, role difficulties, and color vision subscales of the VFQ-25 suggest that the improvements associated with Kalifilcon A DDCLs are not only statistically significant but also meaningful from a patient-centered perspective. Conversely, the small effect sizes for tear film parameters highlight that subjective benefits may not be fully explained by changes in objective measures, underscoring the multifactorial nature of CL comfort.

This study has some limitations that should be acknowledged. The main potential confounding factor is the difference in CL replacement modality between the two groups: one group wore new DDCL (Kalifilcon A DDCL), and the other group wore their habitual reusable CLs with a daily cleaning regimen. While this difference is unlikely to have affected the objective outcomes related to tear film stability and ocular surface health, it may have contributed to the subjective improvements reported with the DDCL. The daily cleaning requirement could have had a negative psychological effect, reducing perceived satisfaction and comfort. Additionally, the cleaning routine itself may have affected ocular surface parameters. Another limitation is that this study was conducted at a single center over a relatively short follow-up period (one month per CL type), which may not reflect long-term outcomes or be applicable to all CL users. Additionally, only symptomatic monthly replacement CL wearers were included, so the results may not apply to asymptomatic users or users of other CL types. Future studies should address these limitations by designing trials in which all study arms use new DDCLs and include the participants’ habitual CL material as a daily disposable version, when available. This would control for the cleaning factor and help determine whether the observed advantages are truly attributable to the Kalifilcon A material. Additionally, similar studies comparing different daily disposable materials could establish whether the observed benefits are unique to Kalifilcon A or extend to other CL designs. Longer-term follow-up and multicenter trials with more diverse populations would also strengthen the evidence base. Finally, while the cleaning routine of monthly CLs could represent a potential confounder, participants were specifically asked to continue with their habitual solution to avoid introducing an additional variable into this study.

In summary, the present study demonstrates that the refitting of symptomatic CL wearers with Kalifilcon A DDCLs leads to a significant reduction in symptomatology, improved tear film stability, and enhanced vision-related quality of life. These findings highlight the potential of Kalifilcon A DDCLs in alleviating discomfort and reducing the risk of CL dropout, a pivotal challenge in clinical practice. However, results should not be generalized to all DDCLs, as comfort is influenced by CL material and mechanical properties, and some DDCLs may not provide similar benefits. Of particular note is the fact that the observed improvements could not be attributed to a placebo effect, thereby underscoring the clinical significance of material selection in optimizing CL performance. Future research should explore the long-term effects of such interventions and evaluate their efficacy across broader patient populations. Furthermore, additional studies should aim to elucidate the underlying mechanisms driving these improvements, particularly regarding the role of tear film stability and ocular surface changes in symptom relief. The elucidation of these factors may contribute to the refinement of clinical guidelines and the development of targeted strategies for the management of symptomatic CL wearers at risk of discontinuation.

## Figures and Tables

**Table 1:** Demographic and baseline information.

	Age	27.1 ± 8.4
	Sex	18 (23%) Male
		61 (77%) Female
	CLDQ-8 score	18.2 ± 4.8
	Subjective satisfaction with CL test score	7.8 ± 0.9
	VQF-25 test score	75.8 ± 8.0
	TFSQ	0.4 ± 0.2
	Area TFSQ	37.9 ± 19.4
	TBUT	3.7 ± 2.5
Ocular health (Efron Grading Scales)	Blepharitis	0.5 ± 0.1
	Meibomian gland dysfunction	0.3 ± 0.1
	Superior limbic keratoconjunctivitis	0 ± 0
	Corneal infiltrates	0 ± 0
	Corneal ulcer	0 ± 0
	Endothelial polymegethism	0 ± 0
	Endothelial blebs	0 ± 0
	Corneal distortion	0 ± 0
	Conjunctival redness	1.5 ± 0.2
	Limbal redness	1.3 ± 0.3
	Corneal neovascularization	0.4 ± 0.2
	Epithelial microcysts	0 ± 0
	Corneal oedema	0.5 ± 0.1
	Corneal staining	0 ± 0
	Conjunctival staining	0.5 ± 0.2
	Papillary conjunctivitis	0 ± 0

CLs = contact lenses; TBUT = tear break-up time; TFSQ = tear film surface quality; A-TFSQ = tear film surface area.

## Data Availability

Data is contained within the article.
